# Bortezomib administration is a risk factor associated with the development of tumor lysis syndrome in male patients with multiple myeloma: a retrospective study

**DOI:** 10.1186/s12885-020-07592-9

**Published:** 2020-11-17

**Authors:** Masahiro Kondo, Yuji Hotta, Karen Yamauchi, Akimasa Sanagawa, Hirokazu Komatsu, Shinsuke Iida, Kazunori Kimura

**Affiliations:** 1 Department of Pharmacy, Nagoya City University Hospital, 1-Kawasumi, Mizuho-cho, Mizuho-ku, Nagoya, Aichi 467-8602 Japan; 2 Department of Hospital Pharmacy, Graduate School of Pharmaceutical Sciences, Nagoya City University, 3-1, Tanabe dori, Mizuho ku, Aichi 467-8603 Nagoya, Japan; 3 Department of Hematology and Oncology, Nagoya City University Graduate School of Medical Sciences, 1-Kawasumi, Mizuho-cho, Mizuho-ku, Nagoya, Aichi 467-8602 Japan; 4 Department of Clinical Pharmaceutics, Nagoya City University Graduate School of Medical Sciences, 1-Kawasumi, Mizuho-cho, Mizuho-ku, Nagoya, Aichi 467-8602 Japan

**Keywords:** Tumor lysis syndrome, Multiple myeloma, Risk factor, Bortezomib, Male

## Abstract

**Background:**

Novel agents such as proteasome inhibitors have been developed for several years to treat multiple myeloma. Although multiple myeloma is a low-risk disease for developing tumor lysis syndrome (TLS), treatment with these novel therapies might increase TLS risk. Previous studies, mostly case reports or case series, have reported bortezomib-induced TLS in patients with multiple myeloma. This study aimed to investigate risk factors associated with TLS development in multiple myeloma patients.

**Methods:**

We retrospectively investigated incidences of laboratory and clinical TLS (LTLS and CTLS, respectively) in patients who received primary therapy for treatment-naive, symptomatic multiple myeloma between May 2007 and January 2018. We used multivariate logistic regression analyses to evaluate the associations between TLS and several parameters previously reported to be associated with increased risk.

**Results:**

This study included 210 patients with multiple myeloma, of which ten (4.8%) had LTLS and seven (3.3%) had CTLS. The characteristics of the administered anticancer or prophylactic antihyperuricemic agents were similar between patients with and without TLS. Multivariate analyses revealed that TLS was most strongly associated with bortezomib-containing therapy (odds ratio = 3.40, *P* = 0.069), followed by male sex (odds ratio = 2.29, *P* = 0.153). In a subgroup analysis focused on men, treatment with bortezomib-containing therapy was significantly associated with increased risk of TLS (odds ratio = 8.51, *P* = 0.046).

**Conclusion:**

In the present study, we investigated the risk factors associated with TLS development in 210 multiple myeloma patients, which, to the best of our knowledge, is the largest number of patients reported to date. Furthermore, this study is the first to evaluate TLS risk factors in MM by adjusting for the effects of potential confounding factors in patients’ backgrounds. Consequently, we found that bortezomib-containing therapy increases the risk of TLS in male patients with multiple myeloma. TLS risk should be evaluated further in low-risk diseases such as multiple myeloma, since a significant number of novel therapies can achieve high antitumor responses.

**Supplementary Information:**

The online version contains supplementary material available at 10.1186/s12885-020-07592-9.

## Background

Tumor lysis syndrome (TLS) is a group of metabolic disorders that involve the massive and abrupt release of cellular components into the blood after the lysis of malignant cells. This occurs following the initiation of cancer treatments, which include the administration of anticancer agents. The release of intracellular components can cause hyperuricemia, hyperkalemia, hyperphosphatemia, and hypocalcemia, which can lead to renal failure, arrhythmias, seizures, and even death [1–6]. In 2004, TLS was subdivided into laboratory TLS (LTLS) including only laboratory data abnormalities such as uric acid, potassium, phosphorus, or calcium, and clinical TLS (CTLS) including both LTLS and clinically important sequelae mentioned above, according to the Cairo–Bishop definition [2]. Thereafter, in 2010, TLS was redefined by an international TLS expert panel [3], which excluded laboratory data on abnormalities in serum calcium. This definition is one of the most recently reported, which is globally accepted and adopted in some of the clinical guidelines for TLS, including the Japanese guidelines. Conversely, the other newer definition of TLS, which aimed at improving the Cairo–Bishop definition, dates back to 2011 [7]. Currently, there are various definitions for TLS.

After the early recognition of patients at risk of TLS, the risk is first evaluated based on malignant disease types and then adjusted according to the known risk factors for each patient. Patients are finally classified as having a high, intermediate, or low risk of developing TLS. In high-risk cases, aggressive prophylactic administration is recommended to treat TLS, such as hydration, allopurinol, febuxostat, or rasburicase (recombinant urate oxidase) [2, 3, 8, 9].

Multiple myeloma (MM) is a malignant neoplasm of plasma cells that accumulates in bone marrow [10]. Regarding TLS risk, MM is currently classified as a low-risk disease, as it is primarily associated with relatively slow cell proliferation rate in the majority of MM cases, excluding the high-risk ones [3]. Over the last two decades, MM treatment has been rapidly evolving with the development of newer generations of medicines, after incorporating of immunomodulatory drug (such as thalidomide) in 1998 and the first proteasome inhibitor (bortezomib: Bor) in 2004 in the European Union [10, 11]. A phase III study of patients with newly diagnosed, untreated MM revealed that Bor plus conventional chemotherapy was superior to chemotherapy alone in terms of both survival and tumor response [12]. Therefore, Bor is an effective treatment for MM, but it might also increase TLS risk by enhancing antitumor responses. In fact, there have been some prior reports of Bor-induced TLS in MM patients [13–19]. However, almost all of them were case reports or case series. There is consequently little information on the effects of Bor on the development of TLS in MM patients; moreover, unknown factors that can increase TLS risk might exist. Given these deficiencies in our current understanding of TLS, we conducted a retrospective study to investigate risk factors for TLS development, including Bor-containing therapy (Bor-CT), in MM patients who received chemotherapy as the primary treatment.

## Methods

### Patients and controls

This study was approved by the ethical review board at the Nagoya City University Graduate School of Medical Sciences (approval number: 60-17-0010). We retrospectively reviewed the medical records of patients who commenced primary treatment for untreated, symptomatic MM at Nagoya City University Hospital, Japan, between May 2007 and January 2018. We excluded patients who did not complete their primary treatment at Nagoya City University Hospital. We also excluded patients who showed laboratory abnormalities that met the criteria for LTLS before and at the commencement of primary treatment, those who underwent dialysis before commencing the primary treatment, and those who had received investigational agents as primary treatments.

The observational period for each patient was from the commencement of primary treatment to the end of the final day of that treatment cycle; data from all completed treatment cycles during this period were used. After the completion of one cycle, if a subsequent cycle did not commence within the timeframe of one treatment cycle, then previously completed cycle was considered the final cycle.

### Diagnosis and evaluation of TLS

We investigated the incidences of LTLS and CTLS that developed during each treatment cycle in the primary treatment period. In this study, TLS was diagnosed according to the modified criteria of the prior definition by Cairo–Bishop et al. (2004), the international TLS expert panel (2010), and Howard et al. (2011) [2, 3, 7]. In brief, LTLS was diagnosed if the serum levels of two or more measurements of uric acid, potassium, or phosphorus were beyond the upper limit of their normal ranges. The diagnosis of CTLS required the presence of LTLS in addition to one or more of the following clinical complications: renal insufficiency (serum creatinine (SCr) ≥ 1.5× upper normal limit), cardiac arrhythmia/sudden death, or seizures.

All clinical variables were collected from patients’ medical records held by the Nagoya City University Hospital. The following data were used to investigate the incidence of TLS or the risk factors for the development of TLS: age, sex, body mass index (BMI) and clinical stage according to the International Staging System (ISS) for Multiple Myeloma, disease type or cytogenetic abnormalities of MM, plasma cells in bone marrow or peripheral blood, presence of extramedullary disease, serum or urine free light chain, serum lactate dehydrogenase (LDH), SCr, estimated glomerular filtration rate (eGFR), uric acid, potassium, phosphorus, calcium, β2 microglobulin, serum or urine albumin, types or number of cycles of therapy regimens, durations of primary treatment, administration of antihyperuricemic agents or infusion volume for hydration, or administration of alkalization agent (sodium bicarbonate) that were initiated before the primary treatment of MM.

### Statistical methods and analysis of risk factors for developing TLS

Differences in characteristics between patients with or without TLS for the administration of anticancer agents or supportive therapy agents, such as antihyperuricemic agents, hydration, and urine alkalization agents, were analyzed by Fisher’s exact test or the Chi-squared test for categorical data and by Student’s t-test or the Mann–Whitney U-test for continuous data. We performed univariate analysis using logistic regression models to assess the association between TLS and each factor that had been hypothesized to be associated with an increased TLS risk in prior reports [1–6, 13, 15]. Then, to adjust for the effects of potential confounding factors in the patients’ backgrounds, multivariate logistic regression analyses were performed using the variables that had been identified as having associations at *P*-value < 0.100 with TLS in the univariate analysis. A P-value < 0.05 was considered statistically significant in this study; all statistical analyses were performed with EZR (Saitama Medical Center, Jichi Medical University, Saitama, Japan), which is a graphical user interface for R (The R Foundation for Statistical Computing, Vienna, Austria) [20].

## Results

### Baseline characteristics of patients and incidence of TLS

This study included 210 patients. The baseline characteristics are summarized in Table 1. The proportion of patients aged ≥65 years was over 60%, and the proportion of men was approximately 50%. As parameters of tumor burden, the mean proportion of plasma cells in the bone marrow was 32.2 ± 22.7%; the proportion of patients with detectable plasma cells in the peripheral blood was 11.9%; the proportion of patients with extramedullary disease confirmed with computed tomography scan was 7.4%; and the mean LDH level was above the upper limit of the normal range. The mean SCr level was 1.19 ± 0.99 mg/dL, and the mean eGFR (61.7 ± 30.3 mL/min/1.73 m^2^) was categorized as “mildly decreased” chronic kidney disease according to the Kidney Disease Improving Global Outcomes (KDIGO) clinical practice guideline criteria [21].

**Table 1:** Baseline characteristics of patients

	No. of patients (%) or Mean ± SD (*n* = 210)
**Age**	
Mean ± SD (years)	67.1 ± 11.1
≥ 65 years (%)	132 (62.9)
**Sex**	
Men	102 (48.6)
**Body mass index**	
Median (range)	22.4 (15.7–38.3)
**ISS stage**	
I	39 (18.6)
II	94 (44.8)
III	77 (36.7)
**Measurable serum M protein**	
IgG	121 (57.6)
IgA	40 (19.1)
IgD	7 (3.3)
Light chain	41 (19.5)
Non-secretory	1 (0.5)
**Involved free light chain**	
Type of free light chain	
kappa	126 (64.6)
lambda	69 (35.4)
Unknown	15
Mean ± SD (mg/L)^a^	
kappa	2494.3 ± 3901.5
lambda	1629.6 ± 3017.4
**Plasma cells in bone marrow (%)** ^b^	
Mean ± SD	32.2 ± 22.7
**Presence of plasma cell in peripheral blood**	
Presence	25 (11.9)
**Presence of extramedullary disease** ^c^	
Presence	13 (7.4)
**Baseline laboratory data (Blood tests)**	
**Lactic dehydrogenase (U/L)**	
Mean ± SD	210.8 ± 132.3
**Creatinine (mg/dL)**	
Mean ± SD	1.19 ± 0.99
**Estimated glomerular filtration rate (mL/min/1.73 m**^**2**^**)**	
Mean ± SD	61.7 ± 30.3
**Uric acid (mg/dL)**	
Mean ± SD	5.8 ± 1.8
**Potassium (mmol/L)**	
Mean ± SD	4.1 ± 0.4
**Phosphorus (mg/dL)** ^d^	
Mean ± SD	3.6 ± 0.7
**β2 microglobulin**	
Mean ± SD (μg/mL)	5.7 ± 3.9
**Albumin**	
Mean ± SD (g/dL)	3.2 ± 0.7
**Calcium**	
Mean ± SD (mg/dL)	9.1 ± 0.9
**Incidence of TLS** ^e^	
Laboratory TLS	10 (4.8)
Clinical TLS	7 (3.3)

*ISS* International Staging System, *TLS* Tumor lysis syndrome, *SD* Standard deviation

^a^Patients with involved free light chain: *n* = 88; the analysis for kappa type included only patients with kappa type of involved free light chain (*n* = 54), and that for lambda type included only patients with lambda type of involved free light chain (*n* = 34)

^b^Patients with involved plasma cells in bone marrow: *n* = 159

^c^Patients with evaluated extramedullary disease shown by computed tomography: *n* = 175

^d^Patients with involved phosphorus: *n* = 119

^e^TLS was diagnosed according to the modified criteria of the prior definition during each treatment cycle in the period of primary treatment [2, 3, 7]

Seventeen patients had developed TLS, from which ten (4.8% of the total) had developed LTLS, and seven (3.3% of the total) were diagnosed with CTLS (Table 1) due to elevated SCr levels. In seven patients with CTLS, four patients met the criteria of myeloma cast nephropathy (CN) at the initiation of primary therapy according to prior reports [22]. No CTLS patients died or were referred for dialysis during the treatment period. In patients with TLS, 11 were in-hospital stay, while six were outpatients. No outpatients needed to be hospitalized for TLS treatment. Out of these six outpatients, five had LTLS with no clinical sequelae and were not worsened despite continuing MM treatment. One remaining patient was given CTLS diagnosis with elevated SCr levels, and was closely monitored as an outpatient receiving febuxostat against hyperuricemia. Renal function for this patient was improved without hospitalization. In the seven patients with CTLS, three and four patients developed CTLS during the first and second treatment cycles, respectively. Moreover, focusing on the timeframe during which CTLS developed in the treatment cycle, three patients developed CTLS within 7 days of commencing the therapy cycle, whereas four patients developed CTLS beyond 8 days.

### Administration of anticancer agents and supportive therapy agents

Anticancer agents and supportive therapy agents for TLS prophylaxis that were administered prior to the commencement of the primary treatment for MM are shown in Table 2 (A). The median number of therapy cycles and duration of the primary treatment were similar between patients who developed and did not develop TLS. The proportion of patients who had their delivered dose reduced from their originally planned dose was also not different between the patients with and without TLS. Regarding the prophylactic measures against the development of TLS, the administration of antihyperuricemic agents, such as allopurinol / febuxostat or rasburicase, hydration, and urine alkalization by sodium bicarbonate agent were used in a nonuniform manner based on physicians’ discretion for each patient. The proportion of patients who were administered antihyperuricemic agents before the initiation of the primary treatment was 34.8%; the proportion of patients who received these agents in this manner was similar between patients who did and did not develop TLS. Out of the 210 patients, 42.4% received hydration which was defined as the average volume over 1000 mL/day for the first 8 days of commencing the primary MM treatment. Moreover, 62.4% received urine alkalization using sodium bicarbonate agent at the start of the primary treatment. The proportion of patients received either hydration or urine alkalization was similar between patients with or without TLS.

**Table 2:** The administration of anticancer agents and supportive therapy agents, and the development of TLS by type of therapy

		Total (***n*** = 210)	Developed TLS (***n*** = 17)	No TLS (***n*** = 193)	***P***-value
(A) **Administration of anticancer agents andsupportive therapy agents for TLS prophylaxis**					
**Anti-cancer agents**					
Number of therapy cycles	Median (Range)				
		3 (1–59)	3 (1–11)	3 (1–59)	0.887
Duration of treatment (days)	Mean ± SD				
		150.6 ± 177.4	143.0 ± 119.8	151.3 ± 181.9	0.854
Dose reduction during treatment	No. (%)				
		115 (54.8)	13 (76.5)	102 (52.8)	0.076
**Antihyperuricemic agents**					
Yes ^a^	No. (%)	73 (34.8)	7 (41.2)	66 (34.2)	0.600
Allopurinol/ febuxostat		72	7	65	
Rasburicase		1	0	1	
**Hydration**					
Yes ^b^	No. (%)	89 (42.4)	8 (47.1)	81 (42.0)	0.880
**Urine alkalization**					
Yes ^c^	No. (%)	131 (62.4)	12 (80.0)	119 (77.8)	1.000
(B) **The development of TLS by type of therapy**					
Bortezomib-containing therapy	No. (%)	130 (61.9)	14 (82.4)	116 (60.1)	0.115 ^e^
Intravenously		29	2	27	
Subcutaneously		101	12	89	
Therapy without Bortezomib ^d^		80 (38.1)	3 (17.6)	77 (39.9)	

*P*-values were determined by Mann-Whitney U-test for number of therapy cycles, Fisher’s exact test for categorical variables, and Student’s t-test for continuous variables

*SD* Standard deviation, *TLS* Tumor lysis syndrome

^a^Defined as being administered prior to the commencement of the primary treatment for multiple myeloma

^b^Defined as the average volume over 1000 mL / day for eight days (from one day before to 7 days after the initiation of multiple myeloma primary treatment)

^c^Defined as being administered sodium bicarbonate agent before the initiation of multiple myeloma primary treatment

^d^Therapies that did not include bortezomib

^e^Comparison between bortezomib-containing therapy and therapy without bortezomib

The number of patients treated with Bor-CT and other therapies that did not include Bor (therapy without Bor) was 130 (61.9%) and 80 (38.1%), respectively (Table 2 (B)). The incidence of TLS in patients treated with Bor-CT had a higher tendency than in those treated with therapy without Bor. Furthermore, six of the seven patients who developed CTLS were also among those who had been treated with Bor-CT. TLS incidence in patients who have received Bor-CT or therapy without Bor is shown in Supplementary Table S1. Treatment regimens in patients treated with Bor-CT were as follows: the Bor plus dexamethasone and melphalan plus prednisolone with Bor regimens were administered to 54.6 and 40.8% of patients, respectively. TLS incidence was not high in specific regimen, and in patients who received triplet regimen, TLS incidence was not higher than those who received doublet regimen (Supplementary Table S2). Regarding bortezomib administration route in the 130 patients who underwent Bor-CT, 29 patients were administered intravenously while 101 subcutaneously; the proportion of patients who developed TLS was similar between both administration routes (Supplementary Table S3). The treatment regimens in patients receiving therapy without Bor were as follows, vincristine plus doxorubicin with dexamethasone, melphalan plus prednisolone, and lenalidomide plus dexamethasone regimens were administered to 46.9, 33.3, and 9.9% of patients, respectively. No patient was treated with carfilzomib, ixazomib, daratumumab, or pomalidomide.

### Analysis of risk factors for developing TLS

Univariate analysis using an unadjusted logistic regression model (crude model) revealed a significant association with high pretreatment SCr levels. This analysis also revealed associations at *P* < 0.100 between TLS and male sex, Bor-CT, ISS stage III, and high pretreatment uric acid levels (Table 3). A high pretreatment level of serum potassium or phosphorus, one of the criteria for the diagnosis of TLS, was not included as an explanatory variable in this analysis, as only six and three patients showed evidence of abnormal serum potassium and phosphorus, respectively, prior to treatment initiation. Additionally, the presence of extramedullary disease was not included in this analysis, since no TLS patients had any extramedullary disease prior to initiating MM primary treatment. The model that was adjusted for age and sex (Model 1) also exhibited similar tendencies as the crude model. The multivariate model (Model 2), which was adjusted for the variables having *P*-values < 0.100 in the crude model, revealed a trend between Bor-CT and the TLS [odds ratio (OR) = 3.40, *P* = 0.069] and that between male sex and TLS development (OR = 2.29, *P* = 0.153). Then, we performed subgroup analyses focusing on sex. In the subgroup analyses of male and female patients (Table 4), Bor-CT was significantly associated with the development of TLS only in males, after adjusting for multiple variables using Model 2 on Table 3 (OR = 8.51, *P* = 0.046).

**Table 3:** Odds ratios and 95% confidence intervals for incidence of TLS using logistic regression models

Variables	Crude model		Model 1		Model 2	
	OR (95% CI)	***P***-value	OR (95% CI)	***P***-value	OR (95% CI)	***P***-value
Age ≥ 65 years	0.83 (0.30–2.28)	0.720	–	–	–	–
Male sex	2.75 (0.93–8.10)	0.067	–	–	2.29 (0.74–7.12)	0.153
BMI > 22.4 (median value on study population)	0.73 (0.26–2.06)	0.559	0.58 (0.20–1.67)	0.310	–	–
Bortezomib-containing therapy	3.10 (0.86–11.10)	0.083	3.11 (0.86–11.30)	0.084	3.40 (0.91–12.70)	0.069
Route for bortezomib administration (Intravenously)	1.82 (0.38–8.64)	0.451	1.92 (0.39–9.37)	0.422	–	–
ISS stage III	2.69 (0.98–7.38)	0.055	2.48 (0.88–6.94)	0.085	1.67 (0.50–5.59)	0.409
β2 microglobulin > ULN	0.79 (0.09–6.76)	0.831	0.60 (0.06–5.75)	0.661	–	–
Serum albumin > ULN	1.57 (0.42–5.90)	0.501	1.32 (0.33–5.33)	0.698	–	–
Lactic dehydrogenase level > ULN	0.93 (0.29–2.99)	0.902	0.91 (0.28–2.96)	0.877	–	–
Pretreatment SCr level > ULN	3.32 (1.14–9.09)	0.027	3.20 (1.12–9.19)	0.030	2.09 (0.61–7.16)	0.241
Pretreatment serum uric acid level > ULN	2.56 (0.92–7.14)	0.072	2.11 (0.73–6.08)	0.166	1.71 (0.56–5.20)	0.344
Serum calcium > ULN (presence of hypercalcemia)	1.47 (0.31–6.99)	0.631	1.19 (0.24–5.81)	0.833	–	–
Plasma cells in bone marrow (%)	1.00 (0.97–1.03)	0.956	1.00 (0.97–1.03)	0.990	–	–
Presence of plasma cell in peripheral blood	0.99 (0.21–4.59)	0.985	0.94 (0.20–1.45)	0.936	–	–
Serum free light chain ratio > 1000	1.14 (0.14–9.51)	0.901	0.96 (0.11–8.40)	0.969	–	–
Hydration ^a^	1.23 (0.46–3.32)	0.684	1.20 (0.42–3.45)	0.729	–	–
Urine alkalization	1.14 (0.31–4.28)	0.843	1.07 (0.28–4.16)	0.919	–	–

Model 1: Adjusted for age and sex, Model 2: Adjusted for the variables that had *P*-values < 0.100 in a crude model

*TLS* Tumor lysis syndrome, *OR* Odds ratio, *CI* Confidence interval, *ISS* International Staging System, *SCr* serum creatinine, *BMI* Body mass index, *ULN* Upper limit of normal range

^a^Defined as the average infusion volume over 1000 mL / day for eight days (from one day before to 7 days after the initiation of multiple myeloma primary treatment)

**Table 4:** Associations between laboratory TLS and bortezomib-containing therapy in patients with multiple myeloma by sex (multivariate analysis)

	No. of patients with TLS/total No. (%)	OR (95% CI)		***P***-value
Female (*n* = 108)				
Therapy without bortezomib	2/42 (4.8)	1.00	(reference)	
Bortezomib-containing therapy	3/66 (4.5)	0.77	(0.09–6.10)	0.804 ^a^
Male (*n* = 102)				
Therapy without bortezomib	1/38 (2.6)	1.00	(reference)	
Bortezomib-containing therapy	11/64 (17.2)	8.51	(1.04–69.60)	0.046 ^a^

*TLS* Tumor lysis syndrome, *OR* Odds ratio, *CI* Confidence interval

^a^ Multivariable adjusted for ISS stage 3, pretreatment serum creatinine level (> upper limit of normal range), and pretreatment serum uric acid level (> upper limit of normal range)

## Discussion

In the present study, we found that Bor-CT is likely to increase TLS risk in MM, particularly among men. MM is always classified as a low-risk disease for developing TLS, regardless of the treatment strategy. However, the type of therapy is a known TLS risk factor [1–6]. TLS risk for chronic lymphocytic leukemia (CLL) is classified as low; however, the risk increases to an intermediate level when targeted therapies (rituximab) or purine analogues (fludarabine) are used [3]. Moreover, in CLL, a higher incidence of TLS development has been reported in patients treated with venetoclax (highly selective inhibitor of BCL2) [23].

In this study, we defined the duration for the diagnosis of TLS as the period of each treatment cycle during primary treatment, although this is different from the criteria of the prior definitions [2, 3, 7], which was defined as the duration within 3 days before or up to 7 days after therapy initiation [3]. Our changes are based on two reasons for the evaluation of treatment-related TLS. The first is to avoid missing TLS cases that develop > 8 days after the initiation of therapy, since some prior case reports have described Bor-induced TLS that developed beyond 8 days [13, 15, 16]. In fact, in our study, we did identify such patients (11 cases). The second reason is to exclude spontaneous TLS that develops before initiating cancer therapy. The baseline blood levels of uric acid, potassium, and phosphorus can spontaneously increase in patients with renal insufficiency. Thus, as such patients could be diagnosed with TLS, we excluded patients who already had laboratory abnormalities that met the criteria for LTLS at the initiation of MM treatment and defined the aforementioned diagnosis period for TLS.

Two prior retrospective studies with small sample sizes investigated the development of TLS in MM patients [18, 19]. These reports described the associations between TLS and the several other factors. However, both prior reports did not use multivariate analysis to adjust for the effects of potential confounding factors. Moreover, in previous studies, the incidence of CTLS was found to be more than approximately four- to five-fold higher (12.7% [18] and 17.2% [19], respectively) than the present study (3.3%). These discrepancies might be due to the abovementioned differences in the TLS diagnosis period, or all or part of the study populations in these prior studies included recurrent/refractory MM (RRMM) patients, whereas our study included only newly diagnosed, untreated patients. The evaluation of treatment-induced TLS might be difficult in cohorts that include both untreated and RRMM because tumor responses generally would be different between untreated and recurrent/refractory cases or among RRMM patients who had varying pretreatment histories [12, 24].

Interestingly, our multivariate analysis revealed that the risk of TLS is increased by Bor-CT only in males. Similarly, a prior retrospective study investigating the risk factors for TLS in patients with acute myelogenous leukemia indicated a significant association between LTLS and the male sex [25]. That report hypothesized that the sex difference in uric acid levels before the commencement of cancer therapy might account for the increase in TLS risk in men, since uric acid levels are generally higher in males than in females [26]. However, our multivariate analysis revealed no significant association between TLS and high pretreatment uric acid levels. Thus, the increase in TLS risk in males cannot be fully explained by only baseline uric acid levels. In contrast, the previous report also suggested that sex might be a surrogate marker for underlying unknown biological differences in the development of TLS. In this regard, a study on Japanese males revealed a strong association between the onset of gout and genetic dysfunction related to urate excretion [27]. A male-specific increase in the risk of developing TLS in patients receiving Bor-CT might also be associated with genetic mechanisms related to urate excretion, given that in the present study, we found that 16 of the 17 patients who developed TLS were diagnosed based on elevated levels of uric acid (data not shown). Hence, TLS risk factors should be evaluated further, as some unknown risk factors potentially exist.

The present study has several limitations, primarily with respect to the study design. The clinical data used to evaluate TLS or pathophysiology and aggressiveness of MM, such as phosphorus levels or information regarding specific myeloma-related common cytogenetic abnormalities (detected by fluorescence in situ hybridization: FISH) and severity of bone disease were missing for some patients. Accordingly, the incidence of TLS associated with phosphorus could have been underestimated, and the effects of cytogenetic abnormalities or the severity of bone disease on TLS were not sufficiently evaluated due to lack of cases tested by FISH or ambiguity with respect to the definition regarding the severity of bone disease. The influence of myeloma CN on the diagnosis of TLS might have also not been accurately evaluated, as most cases analyzed in this study had not received a renal biopsy. Further, some information related to the worsening of CN or renal insufficiency due to factors other than myeloma might have been undetermined, such as the presence of transient dehydration or some concomitant medications. Moreover, the detection power of our study might not have been sufficient based on the sample size. In addition, selection bias could not be avoided; hence, we analyzed all patients who met the criteria for enrollment in this study to reduce bias as much as possible.

## Conclusion

In the present study, we investigated the incidence of treatment-related TLS and the risk factors for developing TLS in 210 MM patients, which, to the best of our knowledge, is the largest number of patients reported to date. Furthermore, this study is the first to evaluate TLS risk factors in MM by adjusting for the effects of potential confounding factors in patients’ backgrounds.

In conclusion, Bor-CT might increase the risk of developing TLS in untreated male MM patients receiving primary therapy. TLS risk should be further evaluated in low-risk diseases such as MM, where the necessity or intensity of prophylactic therapy is not currently established, as an increasing number of novel anticancer therapies can achieve high antitumor responses. Additionally, such evaluations should consider that TLS could develop via the influence of multiple factors that were observed in this study.

## Supplementary Information

**Additional file 1.**

## Data Availability

The datasets used and/or analyzed during the current study are available from the corresponding author on reasonable request.

## Abbreviations

TLS: Tumor lysis syndrome
LTLS: Laboratory tumor lysis syndrome
CTLS: Clinical tumor lysis syndrome
MM: Multiple myeloma
Bor: Bortezomib
Bor-CT: Bor-containing therapy
SCr: Serum creatinine
BMI: Body mass index
ISS: International Staging System
LDH: Lactate dehydrogenase
eGFR: Estimated glomerular filtration rate
CN: Cast nephropathy
OR: Odds ratio
CLL: Chronic lymphocytic leukemia
RRMM: Recurrent/refractory multiple myeloma
