# Supplementary material for: Bortezomib administration is a risk factor associated with the development of tumor lysis syndrome in male patients with multiple myeloma: a retrospective study
Source: BMC Cancer. 2020 Nov 17;20:1117. doi: 10.1186/s12885-020-07592-9 (PMC7672870; doi:10.1186/s12885-020-07592-9)
Supplement: Supplementary file 1 — Additional file 1. [file 12885_2020_7592_MOESM1_ESM.docx]

**Supplementary Tables.**

**Table S1. The incidences of TLS in patients who received bortezomib-containing therapy or therapy without bortezomib**

|  | **No. of patients (%)** | | **P-value** |
| --- | --- | --- | --- |
|  | **Bortezomib-containing therapy**  **(n = 130)** | **Therapy without bortezomib**  **(n = 80)** |  |
| **Developed TLS** | 14 (10.8) ^a^ | 3 (3.8) ^b^ | 0.115 |
| **No TLS** | 116 (89.2) | 77 (96.2) |  |

a: Involved eight laboratory TLS cases with only laboratory data abnormalities and six clinical TLS cases with elevated SCr levels

b: Involved two laboratory TLS cases with only laboratory data abnormalities and one clinical TLS case with an elevated SCr level

TLS: tumor lysis syndrome. SCr: serum creatinine. P-values were determined by Fisher's exact test.

**Table S2. The incidences of TLS by type of bortezomib regimen**

| **Type of bortezomib regimen (n=130)** | | **No. of patients who developed TLS (%)** | | |
| --- | --- | --- | --- | --- |
|  |  | **Total** | **LTLS** | **CTLS** |
| **Doublet regimen** |  |  |  |  |
| BD (bortezomib + dexamethasone) | (n=71) | 8 (11.3) | 3 | 5 |
| **Triplet regimen** |  |  |  |  |
| MPB (melphalan + prednisolone + bortezomib) | (n=53) | 5 (9.4) | 5 | 0 |
| BLd (bortezomib + lenalidomide + dexamethasone) | (n=6) | 1 (16.7) | 0 | 1 |

TLS: tumor lysis syndrome, LTLS: laboratory TLS, CTLS: clinical TLS

**Table S3. Types of route for bortezomib administration**

| **Administration route** | **No. of patients (%)** | | | **P-value** |
| --- | --- | --- | --- | --- |
|  | **Total**  **(n=130)** | **Developed TLS**  **(n=14)** | **No TLS**  **(n=116)** |  |
| **Intravenously** | 29 (22.3) | 2 (14.3) | 27 (23.3) | 0.734 |
| **Subcutaneously** | 101 (77.7) | 12 (85.7) | 89 (76.7) |  |

TLS: tumor lysis syndrome P-values were determined by Fisher's exact test.
